# HIV low‐level viraemia: considerations for prevention and treatment in an era of highly effective and durable antiretroviral therapy regimens

**DOI:** 10.1002/jia2.70085

**Published:** 2026-02-24

**Authors:** Lara Vojnov, Linda‐Gail Bekker, Myron S. Cohen, Andreas Jahn, Diane Havlir, Debrah Boeras, Nathan Ford, Nagalingesawaran Kumarasamy, Laura N. Broyles

**Affiliations:** ^1^ Global Health Impact Group Atlanta Georgia USA; ^2^ Desmond Tutu HIV Centre Department of Medicine University of Cape Town Cape Town South Africa; ^3^ University of North Carolina (UNC) at Chapel Hill Chapel Hill North Carolina USA; ^4^ Department of HIV AIDS Ministry of Health Lilongwe Malawi; ^5^ International Training and Education Center for Health Department for Global Health University of Washington Seattle Washington USA; ^6^ Division of HIV ID and Global Medicine Department of Medicine University of California San Francisco San Francisco California USA; ^7^ World Health Organization Geneva Switzerland; ^8^ CART CRS Voluntary Health Services Chennai India

**Keywords:** HIV, viral suppression, prevention, treatment, sexual HIV transmission, low‐level viraemia

## Abstract

**Introduction:**

Despite increasingly widespread acceptance of the (undetectable = untransmittable) U = U concept, uncertainty remains about the implications of suppressed viral loads (detected but ≤1000 copies/ml, also often referred to as low‐level viraemia) for both sexual HIV transmission and individual patient outcomes.

**Discussion:**

There has been no documented evidence of a transmission event when the index partner had a viral load <200 copies/ml, suggesting zero risk of sexual transmission. Additionally, the risk of sexual transmission when the index partner is taking medication as prescribed and has a viral load that is detectable but suppressed (*≤*1000 copies/ml) is negligible. The clinical implications, including drug resistance development, of persistent low‐level viraemia in people with HIV (PWH) taking dolutegravir‐containing antiretroviral therapy remains limited and relatively unknown; ongoing research and surveillance will be critical to monitor expanded scale‐up of optimized treatment. To support widespread access to treatment monitoring for all PWH, viral load testing should be expanded using any combination of available viral load technologies and sample types, as they can all support the primary objectives of understanding the viral load of PWH, for both prevention and treatment.

**Conclusions:**

PWH who have an undetectable or suppressed viral load should be celebrated and encouraged to maintain their treatment adherence and engagement in care, while ensuring that no remaining barriers exist for them to do so.

## 1 INTRODUCTION

In 2017, the HIV treatment activist group, *i‐base*, published an article promoting the view that a “negligible” risk of HIV transmission should be considered zero risk of transmission [1]. In legal terms, negligible can be understood to be “so small or unimportant as to be not worth considering; insignificant” [2]. The intention was to avoid semantic confusion that could distract attention from the clear advocacy message of U = U (Undetectable = Untransmittable), the statement that sexual HIV transmission does not occur when a person with HIV (PWH) has an undetectable viral load. Multiple organizations and national governments have voiced support for U = U. In a July 2023 policy brief, the World Health Organization (WHO) outlined three viral load thresholds: undetectable (virus not detected), suppressed (detected but ≤1000 copies/ml), and unsuppressed (>1000 copies/ml) and expanded the U = U message by stating that viral loads ≤1000 copies/ml in PWH who are taking medication as prescribed are not associated with sexual transmission of HIV [3].

However, despite increasingly widespread acceptance of the U = U concept, uncertainty remains about the implications of suppressed viral loads (detected but ≤1000 copies/ml) for both sexual HIV transmission and individual patient outcomes. This article addresses key considerations related to these issues and the impact of increasing integrase strand transfer inhibitor (INSTI)‐based antiretroviral therapy (ART) use.

## 2 DISCUSSION

### 2.1 Prevention: The risk of sexual HIV transmission when viral load is undetectable (<200 copies/ml)

There has been no documented evidence of a sexual HIV transmission event when the index partner had a viral load <200 copies/ml. In particular, three randomized‐controlled trials that were designed to assess transmission risk among men who have sex with men not using condoms—Opposites Attract, PARTNER and PARTNER2 [4, 5, 6]—observed no transmission events between the nearly 2500 serodiscordant couples when the index partner had a viral load <200 copies/ml. A fourth study, HPTN052, conducted among heterosexual serodiscordant couples similarly observed no transmission events when the treated individual was stably suppressed by ART [7]. These data formed the basis of many U = U campaigns, solidifying the evidence base that there is zero risk of sexual transmission when a viral load is <200 copies/ml [8, 9].

### 2.2 The risk of sexual HIV transmission when viral load is suppressed (detectable but ≤1000 copies/ml)

The risk of sexual transmission when a viral load is detectable but suppressed (*≤*1000 copies/ml) versus undetectable has been poorly delineated. However, a recent systematic review including nearly 8000 serodiscordant couples with index partner viral loads across the spectrum, including undetectable, concluded that there is a negligible risk of sexual transmission of HIV with viral loads <1000 copies/ml [10]. Documented sexual transmission events have only occurred at higher viral load levels. In an observational study predating the global rollout of ART, 95% of transmission events occurred when the index partner had a viral load >3500 copies/ml [8]. In the flagship multicentre randomized controlled study of heterosexual couples that proved HIV treatment prevents transmission, all transmission events occurred when the index partner had a viral load between 43,000 and 750,000 copies/ml [7]. In four prospective studies that included PWH who had detectable viral loads, no verifiable transmission events occurred when the index partner had a viral load ≤1000 copies/ml [7, 8, 11, 12]. Two potential cases out of nearly 8000 studied had viral loads of ∼615 and ∼850 copies/ml [7, 12], respectively; however, the viral load samples in these cases were drawn more than 50 days prior to the transmission event. Given that viral rebound in cases of treatment interruption or cessation is relatively rapid (median time to viral load of >1000 copies/ml was 22 days in a recent study) [13, 14], the long time interval casts doubt that the viral load remained suppressed (≤1000 copies/ml) at the time of the transmission event. Unfortunately, clinical information for these cases were limited and unavailable, particularly whether the PWH had discontinued medication, fully or partially; therefore, there is a possibility that these viral loads remained at the time of sexual transmission. The longitudinal viral dynamics in PWH with intermittent treatment adherence (as opposed to full interruption of ART) remain relatively unknown. Furthermore, precise quantification of the negligible risk of sexual transmission when a viral load is detectable but suppressed has not yet been generated, yet could be helpful in communication materials.

Additionally, a recent model provides insights into the degree to which low‐level viraemia (defined in the model as 51–999 copies/ml) contributes to the overall epidemic through sexual transmission [15]. Based on available transmission evidence, the model showed that >80% of HIV transmission events occur when the index partner was (1) unaware of their HIV status and thus not on ART or (2) aware of their HIV status, but not on ART. The remaining transmission events in the model occurred when the index partner was on ART but with an unsuppressed viral load (>1000 copies/ml).

### 2.3 Treatment: Persistent low‐level viraemia and the risk to individual health in the INSTI era

Effective ART suppresses viral replication, restores immune function and sustains the health of the individual. Low‐level viraemia can be a result of ART non‐adherence, insufficient drug levels due to drug interactions or malabsorption, incomplete access to treatment and drug resistance. Persons with persistent low‐level viraemia have a higher risk for subsequent virologic failure and drug resistance [16, 17, 18], which can then lead to downstream adverse HIV‐ and non‐HIV‐related co‐morbidities [19]. Risk for these consequences increases with the level of circulating virus. Further, emerging evidence suggests that a single‐time‐point low‐level viraemic result may be a predictor of poor outcomes [20]. Some studies have found an association between low‐level viraemia and increased markers of inflammation and/or immune activation [21, 22]. In persons treated with second‐generation INSTIs (e.g. dolutegravir and bictegravir), low‐level viraemia is less frequent than with other regimens, and the risk for virologic failure and drug resistance in persons with low‐level viraemia is much lower compared to prior regimens [20, 23, 24]. This is likely due to second‐generation INSTI's greater antiviral potency, high genetic barrier to resistance and higher tolerability when given in combination regimens, compared to previous ART regimens. Data on the clinical implications of persistent low‐level viraemia in PWH taking dolutegravir‐containing ART is sparse. One recent analysis found an increased risk of developing any non‐infectious comorbid disease in participants on dolutegravir with low‐level viraemia [19], while another found no association with non‐AIDS events but a higher risk of AIDS events and death [25].

Fortunately, dolutegravir‐based regimens are accessible for low‐ and middle‐income countries (LMICs) for a markedly reduced cost due to major efforts by countries, donors and partners. Over 90% of PWH on ART in LMICs are on dolutegravir‐based regimens [26, 27]. However, recent decisions in the HIV donor landscape have caused major disruptions in treatment services and could jeopardize future access to continuous ART [28].

Drug resistance presenting as low‐level viraemia may be less frequent when the vast majority of PWH are on ART that includes a second‐generation INSTI and have not had access to prior sequential integrase inhibitors. To date, resistance to dolutegravir has remained limited [29, 30, 31, 32, 33]; however, research and surveillance strategies remain in place to monitor possible resistance to this and other commonly used antiretroviral drugs. Initial drug resistance surveys in multiple countries have found high rates (>90%) of viral suppression when PWH are on dolutegravir‐based ART [29, 33]. One survey from Zambia conducted genotype sequencing among adults with HIV who had an unsuppressed viral load (>1000 copies/ml) and found no resistance to dolutegravir. Drug resistance surveys in children and adolescents receiving dolutegravir‐based ART similarly observed no resistance to dolutegravir [33]. Two studies have reported evidence of emerging resistance to dolutegravir in 10 individuals [30, 31]. More recently, the WHO *HIV drug resistance Brief Report 2024* that compiled evidence from surveys performed by CDC and partners, observed an increase in acquisition of resistance to dolutegravir among persons with unsuppressed viral loads when compared to previously reported clinical trial data; however, minimal drug resistance was observed in surveys that included infants and adults initiating or reinitiating ART or among ART‐experienced PWH who switched to a dolutegravir‐based dual or triple drug regimen [33]. Most resistance observed was among PWH who were highly treatment‐experienced with an unsuppressed viral load, or those switched to dolutegravir monotherapy. A 2024 presentation of viraemia and resistance among PWH in Zambia and Malawi switching from a non‐nucleoside reverse transcriptase inhibitor (NNRTI)‐based to dolutegravir‐based ART found that 2 years after switch, the prevalence of viral load >400 copies/ml among the 2388 participants with available samples was 4.7% in Malawi and 1.8% in Zambia [34]. Among those viraemic participants, only two of the 74 samples genotyped had major INSTI mutations. These data are reassuring but highlight the need for continued vigilance along with further investigation of the relationship between INSTI genotypic resistance (i.e. the presence of INSTI mutations) and the impact of those mutations on a drug's ability to maintain virologic control. The growing body of knowledge on HIV drug resistance has repeatedly shown that the relationship between genotypic mutations and drug efficacy is highly nuanced and complex [35].

Because of the high barrier to resistance among second‐generation INSTIs, intermittent treatment adherence is likely a considerable contributor to low‐level viraemia (and later virologic failure) in PWH taking these regimens, underscoring the importance of adherence support. Suggested guidance on actions for virologic thresholds are included in the recent WHO brief [3]. However, until the risk of low‐level viraemia to individual health is clearer for people taking optimized treatment, continued monitoring and management, such as repeated viral load testing, should be considered to exclude the risk of drug resistance and support achievement of an undetectable viral load.

As scale‐up continues of ART containing dolutegravir, other INSTIs and long‐acting formulations, including dual therapies such as cabotegravir and rilpivirine, additional research and data should be collected and collated to better inform any implications of low‐level viraemia at various thresholds to a PWH's individual health, particularly in a public health context and setting. Furthermore, more research is needed to identify optimal adherence interventions, in general and for specific populations, including those with low‐level viraemia.

### 2.4 Viral load technologies to expand treatment monitoring

Most importantly, it is critical to acknowledge that all viral load technologies measure the viral load at one point in time (i.e. at sample collection) and that the durability of that measurement cannot be assumed. This is especially relevant in LMICs where viral load measurements may occur only annually or even less frequently, and where long turnaround times for return of viral load testing results to providers (and ultimately to clients) are the norm. While long‐acting injectable ART regimens are available [36], most current ART regimens require daily dosing, so even an undetectable viral load can increase quickly if interruptions in ART occur due to intermittent treatment adherence, supply shortages, intercurrent illness or other factors. As such, the importance of maintaining client engagement in care, supporting adherence to visits and medications, and addressing barriers to healthcare access cannot be overstated and must be coupled with any treatment monitoring strategy. This also necessitates continued programmatic efforts to provide timely viral load results to providers and clients so that the fidelity and effectiveness of the critical prevention and treatment messages are maximized. Given the very low risk of treatment failure from drug resistance to dolutegravir‐based ART and the limited access to frequent viral load monitoring, health education should emphasize the simple message that consistent adherence makes HIV untransmissible and protects health.

Multiple WHO‐prequalified technologies are available that can accurately measure HIV viral load [37]. Each of these approaches has its advantages. Plasma samples provide the most sensitive results for HIV viral load testing (lowest limits of detection); however, viral load testing using plasma requires that blood samples undergo laboratory processing within 24 hours of sample collection. Point‐of‐care testing devices provide immediate information in clinical settings and are particularly important for PWH who need timely feedback on high viral loads (e.g. pregnant women, children, adolescents and those suspected of treatment failure) [38, 39]. Alternative tests and sample types can enable broader scale‐up and sustainable access to viral load testing that may be preferable in some settings [40]. Near point‐of‐care and alternative sample collection types, such as dried blood spot samples, may have higher limits of detection (the lowest amounts of virus that can be detected 95% of the time) than plasma‐based technologies; however, they do not require significant processing and thus can support more decentralization and greater access to wider populations of PWH.

Further, having a higher limit of detection does not preclude these technologies from supporting treatment programme goals to identify viral load values as undetectable, suppressed and unsuppressed. Two recent systematic reviews have summarized the performance of dried blood spot samples and near point‐of‐care viral load technologies to identify viral load results above or below a variety of viral load thresholds analysed, including undetectable [41, 42]. All available technologies provide accurate results when a viral load is higher than the threshold of choice. In both studies cited above [38, 39], the sensitivity of measuring a viral load >200 copies/ml was greater than 95% compared with laboratory‐based plasma testing. Additionally, the specificity of measuring a viral load <200 copies/ml (including undetectable) was 93–95% for viral load technologies with WHO‐prequalification. Therefore, even when using technologies with higher limits of detection, alternative viral load technologies can still accurately detect low‐level viraemia [37, 40, 41, 42]. Indeed, all WHO‐prequalified viral load technologies are quantitative and can provide viral load test results that are undetectable, suppressed (detectable but ≤1000 copies/ml) and unsuppressed (>1000 copies/ml).

It is also important to recognize that viral load testing uses polymerase chain reaction technology that is known to be inherently variable [43, 44], even using the most sophisticated and accurate reference standards. For example, a viral load of 1000 copies/ml (even if using a plasma specimen and a laboratory‐based assay) has a known and accepted variability in the range of 500–2000 copies/ml, meaning that if the test was repeated, the result would likely be anywhere within that range [43, 44].

Moreover, there is evidence that low‐level viraemia may be less common than reported, with studies suggesting suboptimal laboratory procedures resulted in misclassification of undetectable viral loads as low‐level viraemia in almost 20% of samples [45]. Laboratories should carefully follow manufacturer instructions for use and standard operating procedures to ensure they do not artificially and inappropriately increase the proportion of PWH having low‐level viraemia.

### 2.5 Conclusions: What does this mean for PWH and for public health?

Based on the sexual HIV transmission risk evidence [10], WHO convened an expert group of over 40 stakeholders across the HIV response, including researchers, implementers and civil society, to develop a policy brief on the role of HIV viral suppression in improving individual health and reducing transmission [3]. First and foremost, long‐term, consistent, and positively reinforced treatment adherence and maintenance should be prioritized for the health and wellbeing of all PWH. Furthermore, the consensus view from the expert panel, summarized in the policy brief, is that PWH who have an undetectable viral load using any WHO‐prequalified combination of sample and testing platform, including dried blood spot samples, and continue taking medication as prescribed have zero risk of transmitting HIV to their sexual partner(s). Additionally, PWH who have a suppressed but detectable viral load (≤1000 copies/ml) and are taking medication as prescribed have a negligible chance of transmitting HIV to their sexual partner(s). As such, they should receive encouragement and celebration for reaching this threshold, while positively addressing adherence and exploring other barriers so that they can reach an undetectable viral load.

The available evidence and the WHO‐convened expert group provided clear key messages for PWH, their partners, their providers and the public. The most important of these is that PWH receiving effective ART can now live longer and pursue as healthy a life as people who are HIV negative [46], while also maintaining a healthy and safe sex life. Together, these messages should be provided to PWH, along with their viral load result, in a clear, consistent, positive and celebratory manner. Developing and disseminating these messages along with associated tools will support improved education, act as a motivation to adhere to medication, and reduce stigma, discrimination and criminalization of PWH. Additionally, the evidence together clearly shows that if the global community wants to end HIV as a public health threat, focus should be placed on identifying all PWH and linking them to optimal, suppressive and sustained ART. At a population level, successful, widespread deployment of ART, alongside effective prevention programmes, has significantly impacted and reduced HIV incidence [47, 48, 49].

## COMPETING INTERESTS

The authors declare no competing interests.

### AUTHORS’ CONTRIBUTIONS

LV: Conceived of the commentary, drafted the initial and revised subsequent versions of the manuscript, and finalized for submission.

1

LNB: Conceived of the commentary, supported drafting of the initial and subsequent versions of the manuscript, and approved of submission.

2

L‐GB, MSC, AJ, DH, DB, NF and NK: Extensively reviewed each version, provided feedback and approved of submission.

## Data Availability

Data sharing is not applicable to this article as no datasets were generated or analysed during the current study.
